# Theta–Alpha Dysregulation reveals impaired endogenous cognitive control in adolescent Obsessive–Compulsive disorder

**DOI:** 10.1016/j.nicl.2026.103975

**Published:** 2026-02-20

**Authors:** Sarah Rempel, Adriana Böttcher, Nicole Beyer, Veit Roessner, Christian Beste

**Affiliations:** a Cognitive Neurophysiology, Department of Child and Adolescent Psychiatry, Faculty of Medicine of the TU Dresden 01307 Dresden, Germany; b Department of Child and Adolescent Psychiatry, Faculty of Medicine of the TU Dresden 01307 Dresden, Germany; c German Center for Child and Adolescent Health (DZKJ), Partner site Leipzig/Dresden, Dresden, Germany

**Keywords:** Cognitive flexibility, OCD, Task Switching, Alpha, Theta, EEG

## Abstract

Obsessive–compulsive disorder (OCD) is characterized by rigid thoughts and behaviors that may compromise cognitive flexibility, yet, especially in adolescence results are inconclusive. This is all the more the case concerning the question whether flexibility deficits emerge specifically when endogenous control, internal monitoring, and working-memory–dependent task-set management are required. In an EEG-beamforming study, we examined adolescents with OCD (N = 68) and healthy controls (HC) (N = 73) in a task-switching paradigm with cue-based (exogenous, externally guided) and memory-based (endogenous, internally guided). Behaviorally, both groups showed typical switch-cost patterns, with greater demands in the memory-based block, and no group differences in switch costs. Oscillatory EEG data revealed marked group differences. During cue-based switching, both groups showed preserved pre-target TBA and ABA modulations in frontal–parietal networks, indicating intact externally preparatory control. In contrast, only HC adolescents showed pre-target TBA increases during memory-based switching, suggesting impaired endogenous task-set updating in OCD. Reactive processes further distinguished groups. In the cue-based block, adolescents with OCD showed switch-related ABA reductions in centro-parietal regions, possibly reflecting inefficient task-set stabilization despite intact behavior. In the memory-based block, HCs displayed coordinated TBA–ABA differentiation supporting internally generated updating, whereas the OCD group showed isolated TBA modulation without accompanying ABA effects, indicating disrupted integration of updating and gating mechanisms. Together, these findings show a dissociation between externally and internally guided flexibility in adolescent OCD.

## Highlights

• Healthy controls and adolescents with OCD show similar switch costs, different neural oscillations.
• Memory-based task switching is more demanding than cue-based switching.
• Theta–alpha coupling differentiates externally cued and self-initiated cognitive flexibility.
• Endogenous control is selectively impaired in adolescents with OCD.
• EEG beamforming reveals source-specific oscillatory differences.

## 1 Introduction

Obsessive-compulsive disorder (OCD) affects 1–2 % of children and adolescents (American Psychiatric Association, 2013) and is defined by recurrent intrusive thoughts (obsessions) and/or repetitive behaviors (compulsions) (Abramowitz et al., 2009). These rigid thoughts and behaviors may lead to deficits in cognitive flexibility (Gu et al., 2007), which have frequently been reported (Chamberlain et al., 2007, Chamberlain et al., 2006, Eng et al., 2015, Gruner and Pittenger, 2017, Meiran et al., 2011, Remijnse et al., 2013, Snyder et al., 2015, Watkins et al., 2005). Cognitive flexibility is a central executive function that enables individuals to shift between mental sets, update rules, and reconfigure stimulus–response mappings in a context-appropriate manner (Diamond, 2013). Converging structural and functional imaging work points to abnormalities in fronto–striatal–thalamic circuits in OCD, including prefrontal, basal ganglia, and thalamic regions (Britton et al., 2010, Eng et al., 2015, Gruner and Pittenger, 2017, Gu et al., 2007, Remijnse et al., 2013, Yang et al., 2023), which are essential for adaptive behavior (Shenhav et al., 2024). While executive dysfunction and reduced flexibility are well documented in adult OCD (Chamberlain et al., 2007, Chamberlain et al., 2006, Eng et al., 2015, Gruner and Pittenger, 2017, Meiran et al., 2011, Remijnse et al., 2013, Snyder et al., 2015, Watkins et al., 2005), evidence in children and adolescents is more mixed: Some studies report no apparent behavioral deficits in cognitive flexibility compared to neurotypical individuals (HC) (Britton et al., 2010), yet still reveal aberrant activation in prefrontal regions during executive tasks (Britton et al., 2010, Gu et al., 2007, Vaghi et al., 2017). Given that OCD often emerges or consolidates during adolescence, this developmental period is crucial for identifying neurophysiological markers of cognitive rigidity that may shape long-term trajectories.

To examine cognitive rigidity, task-switching paradigms are widely used tasks (Diamond, 2013, Kiesel et al., 2010, Monsell, 2003). They require individuals to alternate between different task rules, thereby engaging processes of task-set updating, interference resolution, and response reconfiguration. Typically, responses are slower and less accurate on switch trials than on repetition trials, yielding “switch costs” that index efficiency of flexible control (Monsell, 2003). Importantly, task switching can be implemented in conceptually distinct modes: In cue-based (exogenous) task switching, external cues specify which rule is currently relevant. This format primarily taxes reactive control processes: the system must decode the cue, update the task set, and configure the appropriate stimulus–response mapping. In contrast, memory-based (endogenous) task switching requires participants to monitor internally when to switch rules (Gajewski et al., 2011, Petruo et al., 2021, Wendiggensen and Beste, 2023, Wolff et al., 2017c, 2017b, 2018). Here, rule changes are not signaled by external cues but rely on working memory, internal monitoring, and self-initiated control. Memory-based switching, therefore, imposes additional demands on working-memory-related mechanisms and can overstrain processing capacities. Within the context of OCD, this is of particular relevance since *meta*-analytic work suggests that timed working memory may be particularly vulnerable in younger OCD samples (Geller et al., 2018), indicating that early-stage (adolescent) OCD may already involve subtle disturbances in the neural architecture of flexible control, even when overt behavior appears relatively preserved. While previous work in adolescent OCD has already used this distinction to probe the mechanisms of inflexibility (Wolff et al., 2017a), the neural mechanisms are still widely elusive. This is particularly true for oscillatory neural processes, which are fundamental to neural information processing (Beste et al., 2023, Buzsáki et al., 2013, Fries, 2015).

Oscillations in the theta (TBA) and alpha (ABA) bands have been repeatedly linked to adaptive action control (Beste et al., 2023). Frontal-midline TBA increases under conditions of conflict, rule updating, and performance monitoring (Cavanagh and Frank, 2014, Cohen, 2014), and is thought to reflect the recruitment of reactive control processes that adjust behavior when demands change (Cooper et al., 2019, Cooper et al., 2016, Sauseng et al., 2006). More particularly, it has been shown that TBA increases when actions need to be reconfigured (Gholamipourbarogh et al., 2025, Pscherer et al., 2020, Rawish et al., 2024, Talebi et al., 2024). In task switching, higher TBA is typically observed during switch compared to repetition trials, especially before and shortly after target onset, indexing the effortful reconfiguration of task sets (Wendiggensen and Beste, 2023). ABA, in contrast, is closely associated with gating and inhibition of information processing (Bonnefond and Jensen, 2012, Foxe and Snyder, 2011, Klimesch, 2012, Konjusha et al., 2023, Yu et al., 2024). More recently, a broader systematic framework has emphasized that alpha rhythms are a key mechanism by which the brain organizes large-scale networks during action control, stabilizes task-relevant representations, and orchestrates transitions between different control states (Beste et al., 2023). Within this framework, ABA does not simply “turn down” processing but shapes the functional architecture in which other processes operate (especially TBA). Work in task-switching paradigms has shown that, in cue-based contexts, ABA decreases after switch cues relative to repetition cues, reflecting the opening of the system for task-set updating (Cunillera et al., 2012, Sauseng et al., 2006). In memory-based switching, elevated ABA before repetition trials has been interpreted as task-set shielding and protection of the currently relevant rule in working memory (Wendiggensen and Beste, 2023, Wolff et al., 2017c). Recent evidence further suggests that the interplay between ABA and TBA enables the management of perception–action representations during adaptive behavior (Wendiggensen et al., 2023). From this integrated perspective, examining TBA and ABA during both cue-based and memory-based task switching in adolescents with OCD is particularly informative. TBA indexes the extent to which reactive control is recruited when task sets must be updated. In contrast, ABA reflects how effectively task representations are gated and shielded in anticipation of and during rule application. Differences between cue-based and memory-based blocks allow us to probe how these oscillatory mechanisms are modulated when flexibility is primarily externally guided versus when it must be internally generated and maintained in working memory.

In the present study, we therefore combined cue-based and memory-based task switching within a single paradigm in a relatively large sample of adolescents with OCD and matched healthy controls (HC). Using EEG beamforming (Gross et al., 2001), we examined TBA and ABA and their source-localized origins during preparatory (pre-target) and reactive (post-target) phases of task switching. Based on previous behavioral and neurophysiological work, we hypothesized that adolescents with OCD would show changes in switch costs, particularly in the memory-based block, and alterations in TBA and ABA relative HCs, especially in frontal, parietal, and temporal regions implicated in inhibitory control and working-memory-related flexibility. Specifically, fronto-parietal brain regions are consistently recruited during task switching (Periáñez et al., 2024), with frontal regions particularly engaged during endogenous switching (Kim et al., 2012), and theta activity over fronto-parietal sites has been linked to cognitive control demands during task switching paradigms (Cooper et al., 2019). Temporal regions, including medial and middle temporal structures, contribute to working-memory-related flexibility and the integration of task-relevant information (Hawkins and Yonelinas, 2024, Wolff et al., 2018). More specifically, we expected that TBA would increase during switch relative to repetition trials, reflecting reactive control, and that this modulation might be altered in the OCD group. Likewise, we expected ABA to differentiate between switch and repetition trials in ways consistent with attentional gating and task-set stabilization, with potential group differences particularly in parietal regions. Finally, we anticipated that individual variability in TBA and ABA modulation would relate to behavioral switching efficiency.

## 2 Methods

### 2.1 Participants

In this study, we enrolled 73 adolescents from a longitudinal study (Rempel et al., 2023) who had been diagnosed with OCD according to ICD-10 criteria or had suspected OCD with a CY-BOCS score of at least 8 points. We only used the data of the first appointment. We also included a total of 74 HCs aged between 10 and 19 years with new and unpublished data and partially with data from Wolff et al. (Wolff et al., 2017a). More details can be found in the supplement. Exclusion criteria were treatment for other primary psychiatric disorders, neurological or developmental disorders, a history of psychosis, a current severe depressive episode, current substance use disorder, acute suicidal tendencies, or an IQ less than 70 (administered by „Der Zahlen-Verbindungs-Test” [ZVT] (Oswald and Roth, 1987). In the OCD sample, participants were excluded from the analyses after visual inspection of the pre-processed EEG data due to excessive artifacts that could not be corrected (N = 4) and because of outliers in the behavioral data from the task-switching paradigm with an accuracy rate of less than 50 % in more than one condition (N = 1). The final sample consisted of N = 68 participants (M_age_ = 15.3 years ± 2.18; M_IQ_ 101 ± 14.22; 50 % male, CY-BOCS score 17.9 ± 7.3). Two further participants showed heavy artifacts in the later EEG analysis that survived the preprocessing. These N = 2 participants were removed from the subsequent EEG analysis (see methods section). Possible comorbidities in the OCD group were assessed using a semi-structured interview according to DSM-V and current clinical reports, with 23 (33.8 %) in the OCD group having comorbid diagnoses and 25 (36.8 %) taking medication in the final sample. More details can be found in the Supplement (Table S1). In the HC group N = 1 participant was excluded from the analyses after pre-processing the EEG-data after visual inspection of the pre-processed EEG data due to excessive artifacts that could not be corrected. The final HC sample consisted of 73 adolescents (M_age_ = 14.6 years ± 2.32; M_IQ_ 112.57 ± 12.27; 43.8 % male). All participants had normal or corrected-to-normal vision. Three participants in the OCD group and one in the HC group were excluded from the EEG analyses due to poor data quality during the beamforming analyses but were retained in the behavioral analyses.

### 2.2 Task switching paradigm

**Fig. 1:**
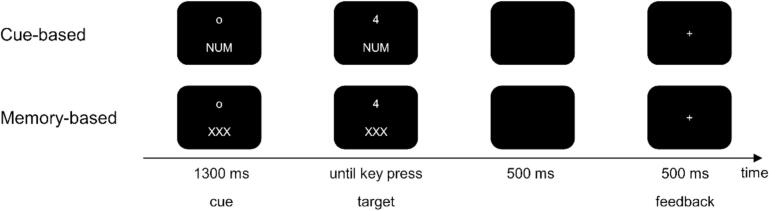
The outline of the task switching paradigm is presented. The cue-based task switching paradigm is shown in the upper part, the memory-based in the lower part of the figure. In the memory-based part, participants have to remember when to switch a rule.

Participants were seated in front of a computer screen to perform a task switching paradigm (Gajewski et al., 2011) according to Wolff et al. (Wolff et al., 2017a). Stimulus presentation, response recording, and EEG triggers were all managed using „Presentation“ software (version 14.9). The visual stimuli comprised numbers 1 through 9 (excluding 5), displayed in a white font against a black screen, with the option to appear in either small or large size. The task was split into two blocks: a cue-based block and a memory-based block. Participants encountered three alternating rules (numeric, parity, and font size) signaled by cues (as depicted in Fig. 1) within the cue-based block. Depending on the specific rule, participants were asked to decide whether the presented number was greater or less than 5 (numeric rule), odd or even (parity rule), or small or large (font-size rule). The rules were randomly switched with an equal likelihood of cue appearance, resulting in a task-switching frequency of 50 %. In the memory-based block, participants were required to switch rules after every third trial based on their memory of the task sequence. The sequence commenced with the numeric task, followed by the parity and font-size tasks, and so forth. Like the cue-based block, the rules were to be applied with an equally distributed probability of 33.3 %, albeit with a task-switching frequency of 33.3 %. The overall paradigm encompassed 768 trials, divided across eight blocks (comprising four cue-based blocks and four memory-based blocks). Participant responses were captured through button presses using their left or right index finger. The response deadline was 2500 ms. Immediate feedback was provided after 500 ms. The response cue interval was set at approximately 1500 ms. All participants completed the cue-based block first, followed by the memory-based block, in a fixed order.

### 2.3 EEG recording and analyses

The EEG data were recorded using QuickAmp and BrainAmp amplifiers with 60 Ag/AgCl electrodes dispersed evenly over an elastic cap (ground electrode at coordinates θ = 58, ϕ = 78; reference electrode at coordinates θ = 90, ϕ = 90) at a 500 Hz sampling. Electrode impedances were kept under 20 kΩ. EEG preprocessing was performed using EEGLAB (Delorme and Makeig, 2004) in Matlab 2021a (The MathWorks Corp.). First, the EEG data was downsampled to 250 Hz. Then, a high-pass filter at 0.5 Hz and a low-pass filter at 40 Hz were applied. Via the cleanline-plugin in EEGlab, line noise at 50 Hz was removed. Subsequently, flat channels were removed and interpolated via a spherical spline interpolation. The data were then re-referenced to average reference. An independent component analysis was applied to remove ocular and cardiac artifacts from the data. To prepare the data for the ICA, a copy of the dataset was rigorously preprocessed using pop_jointprob. An extended infomax algorithm was used for the ICA, and the resulting components were visually inspected regarding their topography, activity time course, and the label estimated via IC-label (Pion-Tonachini et al., 2019). Artifactual components were removed from the data (not the data copy that was rigorously preprocessed for the ICA, but the data created in the average reference step) and the resulting data was epoched for switch and repetition trials separately. No baseline correction was performed, all results reported later are based on relative power differences. Remaining artifactual epochs were removed via pop_jointprob. After the preprocessing, an average of 74.6 ± 11.5 trials remained per condition (cf. 83.77 ± 8.64 trials in the behavior).

The epoched EEG data were imported to the FieldTrip toolbox (Oostenveld et al., 2011) for subsequent analyses. Only trials with correct responses were included. A time–frequency analysis was applied to the single-trial data using Morlet wavelets with a width of 5 Gaussians and a Hanning taper. Upon visual inspection of the time–frequency results, two participants of the OCD sample were excluded due to heavy artifacts that survived the preprocessing. The subsequent analysis was performed with N = 66 subjects in the OCD sample.

For the following analyses, the epochs were divided into a pre-target (−1000 msec until target presentation) and a post-target (0–1000 msec after target presentation) interval. For each time interval and group, cluster-based permutation tests with 1000 Monte-Carlo iterations were used to statistically examine power differences in the alpha (8–12 Hz) and theta (4–7 Hz) frequency band between the switch- and repetition condition in the memory-based and cue-based task blocks, respectively. Cluster-based permutation testing was performed with an alpha level of 0.05 and a cluster level alpha of 0.05. For all contrasts that revealed significant power differences in the cluster-based permutation test, the underlying neuroanatomical sources were retrieved using a beamforming approach. The source reconstruction was done based on the dynamic imaging of coherent sources (DICS) beamformer (Gross et al., 2001). Specifically, via Fast Fourier Transform (FFT), the cross spectral density matrix was calculated. The DICS beamformer was subsequently applied to the appended FFT data and a common spatial filter was calculated. Beamforming was then performed using the MNI-based forward model with a spatial resolution of 5 mm and was applied to the single-subject data for each condition, respectively. The contrast was then established by calculating a ratio, to normalize the difference, reduce bias due to noise and avoid outlier driven effects (Mückschel et al., 2016). The ratio was calculated as follows:

$$r=\frac{{\mathrm{power}}_{cond1}-{\mathrm{power}}_{cond2}}{{\mathrm{power}}_{cond1}+{\mathrm{power}}_{cond2}}$$

Afterwards, in order to describe circumscribed clusters of source activity differences, the ‘Density-Based Spatial Clustering of Applications with Noise’ (DBSCAN) algorithm (Ester et al., 1996) was applied. The 1 % most positive or negative (depending on the direction of the contrast) voxels were included, and only those voxels within neuroanatomical regions (labelled based on the Anatomical labeling atlas) were used. The neighborhood search radius (i.e. the maximum distance between two voxels’ centers to be clustered together) for the DBSCAN algorithm, i.e. the epsilon parameter, was set to 1.5 times the length of the voxel’s edges. The resulting clusters were inspected visually based on the neuroanatomical regions and the number of voxels. To include only reliable spatial clusters, only those containing at least 10 voxels are reported.

### 2.4 Statistics

We used IBM SPSS Statistics (Version 30.0.0.0) to analyze the data. In regards to the task switching paradigm, we eliminated trials with RTs greater than 2,500 ms or less than 100 ms from the remaining trials. Behavioral data were analyzed using mixed effect ANOVA. This model included the within subject factor „Condition” (repeat vs. switch), „Block” (cue-based vs. memory-based) and the between-subject factor „Group” (OCD vs. HC). Greenhouse–Geisser corrections were applied. All post-hoc tests were Bonferroni-corrected.

## 3 Results

### 3.1 Behavioral data

#### 3.1.1 Reaction times and switch costs

**Fig. 2:**
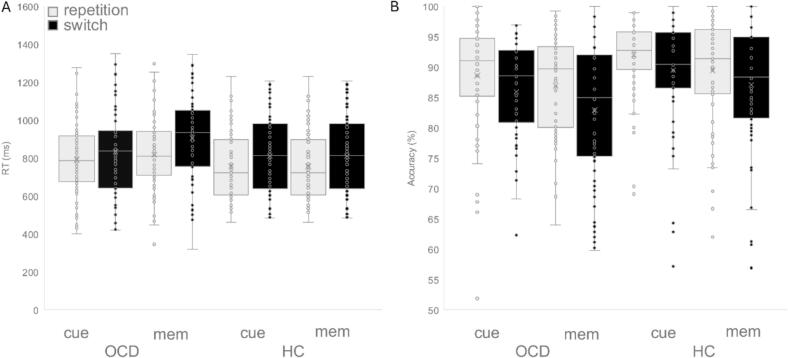
Behavioral data are shownfor the HC and OCD group for the different conditions (switch and repetition) for the cue-based and memory-based block. A: Reaction times. B: Mean accuracy. Within each box, horizontal grey lines denote median values; the „x” represents the mean, boxes extend from the lower quartile Q1 to the upper quartile Q3; whiskers above and below the box indicate maximum and minimum values of the data; dots appear beyond a distance of 1.5 times the interquartile range.

The mixed-effects ANOVA revealed main effects of (i) „Block” [*F*(1,139) = 46.53, *p* < 0.001, *η^2^* = 0.251], indicating increased RTs during the memory-based (854.79 ms ± 16.07) versus cue-based block (799.01 ms ± 16.52), (ii) „Condition” [*F*(1,139) = 169.027, *p* < 0.001, η^2^ = 0.549], indicating increased RTs during the switch (863.28 ms ± 17.05) versus repetition condition (790.52 ms ± 14.92). The effects were further specified by a significant interaction of „Block x Condition” [*F*(1,139) = 35.053, *p* < 0.001, *η^2^* = 0.201], indicating increased RTs during the switch versus repetition condition in both blocks, cue-based (822.66 ms ± 18.02 vs. 775.35 ms ± 15.48, *p* < 0.001) and memory-based (805.68 ms ± 15.46 vs. 903.9 ms ± 17.57, *p* < 0.001). No other effect was observed to be significant (all *p* ≥ 0.178). With respect to switch costs, there was a main effect of „Block” [*F*(1,139) = 35.053, *p* < 0.001, η^2^ = 0.201] showing higher switch costs during the memory-based (98.22 ms ± 7.88) as compared with the cue-based (47.31 ms ± 6.12, *p* < 0.001) block. No other effect was observed to be significant (all *p* ≥ 0.178) (Fig. 2).

#### 3.1.2 Hit accuracy

A mixed-effects ANOVA on accuracy (percentages of hits) yielded a significant main effects of (i) „Block” [*F*(1,139) = 18.126, *p* < 0.001, *η^2^* = 0.115], indicating higher accuracy in the cue-based (89.02 % ± 0.67) than the memory-based block (86.62 % ± 0.8), and (ii) „Condition” [*F*(1,139) = 89.581, *p* < 0.001, *η^2^* = 0.392], indicating higher accuracy during repetition (89.27 % ± 0.64) than during switch trials (86.37 % ± 0.75). There was a between subject effect of „Group” [F(1,139) = 6.355, *p* = 0.013, *η^2^* = 0.044], indicating a higher accuracy in the HC group (89.53 % ± 0.94) compared to OCD group (86.11 % ± 0.96). No interaction was observed to be significant (all *p* ≥ 0.136).

### 3.2 Neurophysiological data

#### 3.2.1 Pre-target interval

**Fig. 3:**
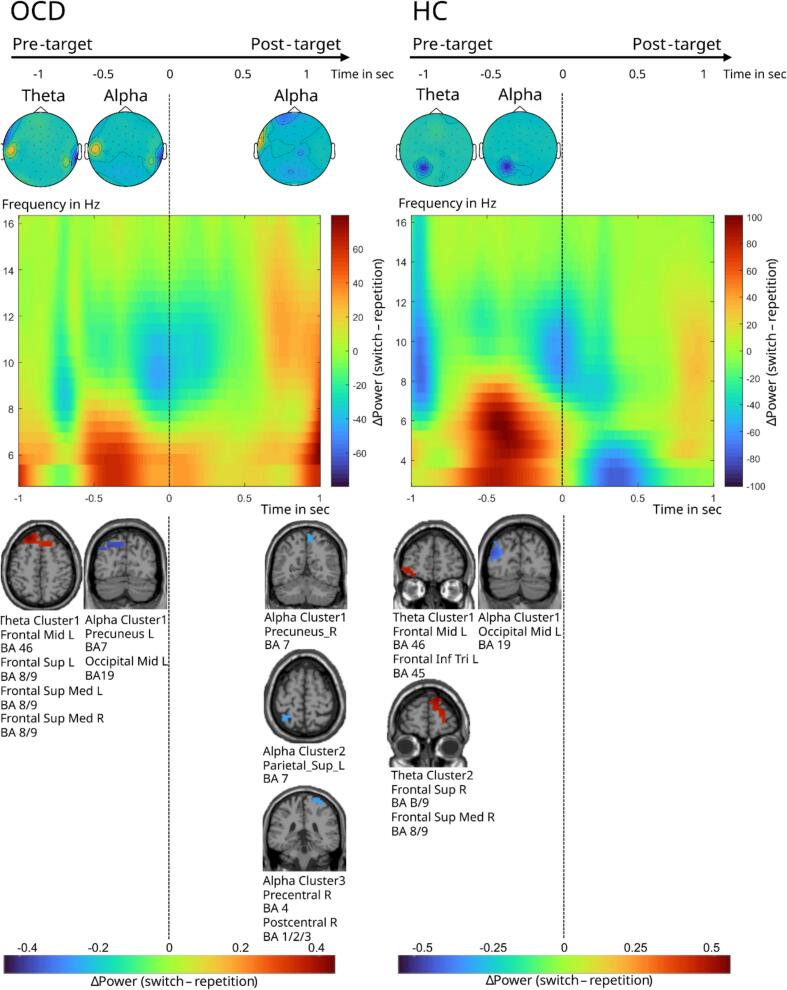
Time–frequency, topographical, and source representations of pre- and post-target theta- and alpha-band activity during switch versus repetition trials in the cue-based block, shown separately for adolescents with OCD and healthy controls.

In the pre-target interval (−1000 ms – 0 ms) in the cue-based block a cluster-based permutation test revealed a significant positive difference in TBA between switch and repetition trials (switch > repetition) in the OCD group (*p* < 0.005) and HC group *(p* < 0.005), indicating lower TBA in the repetition as compared with the switch condition. On the source level, the TBA difference between switch and repetition trials in the OCD group is strongest in one clusters of voxels in the left and right superior frontal gyrus (BA 8/9), in the left and right medial superior frontal gyrus (BA 8/9) and one in the left middle frontal gyrus (BA 46) (Fig. 3) and in the HC group in two clusters of voxels: one cluster in the left middle frontal gyrus (BA 46) and the left pars triangularis (inferior frontal gyrus) (BA 45), and another cluster is in the right superior frontal gyrus (BA 8/9) and the right medial superior frontal gyrus (BA 8/9) (Fig. 3).

Additionally, in the cue-based block there was a significant negative difference in ABA between switch and repetition trials (switch < repetition) in the OCD group *(p* < 0.005) and HC group *(p* < 0.005), indicating lower ABA in the switch as compared with the repetition condition. On the source level, the ABA difference between switch and repetition trials in the OCD group is strongest in one cluster of voxels in the left precuneus (BA 7) and in the left middle occipital gyrus (BA 19) (Fig. 3) and in the HC group in one cluster of voxels in the left middle occipital gyrus (BA 19) (Fig. 3).

**Fig. 4:**
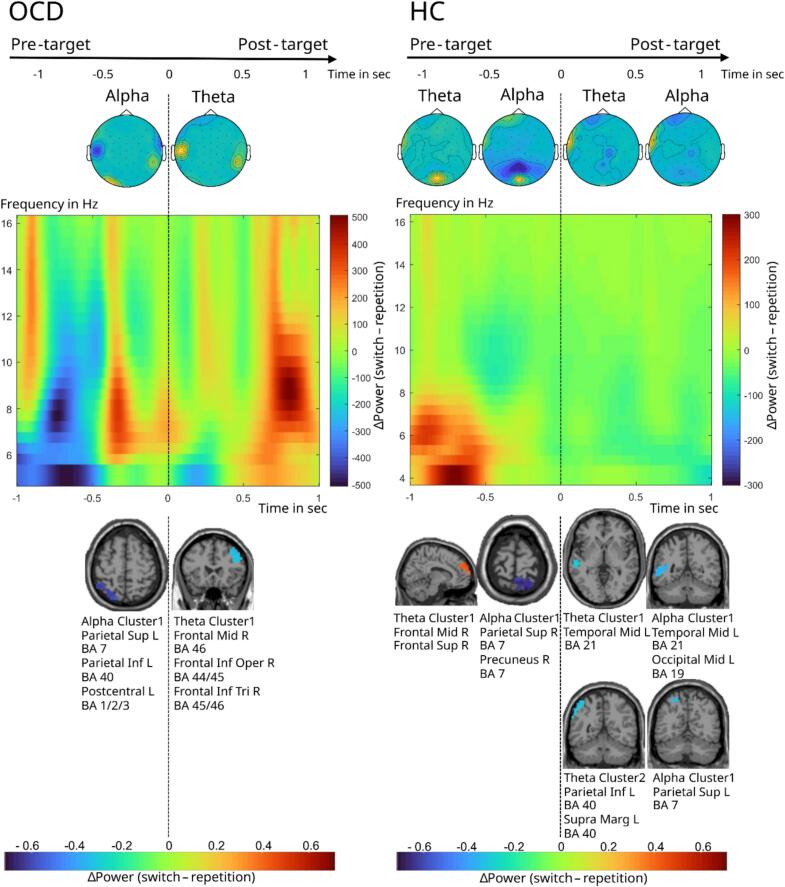
Time–frequency, topographical, and source representations of pre- and post-target theta- and alpha-band activity during switch versus repetition trials in the memory-based block, shown separately for adolescents with OCD and healthy controls.

In the memory-based block a cluster-based permutation test revealed a significant positive difference in TBA between switch and repetition trials (switch > repetition) in the HC group *(p* < 0.05), indicating lower TBA in the repetition as compared with the switch condition. On the source level, TBA difference between switch and repetition trials in the HC group is strongest in one cluster of voxels in the right medial superior frontal gyrus (BA 8/9) and right superior frontal gyrus (BA 8/9) (Fig. 4). Additionally, there was a significant negative difference in ABA between switch and repetition trials (switch < repetition) in the OCD group *(p* < 0.005) and HC group *(p* < 0.005), indicating lower ABA in the switch as compared with the repetition condition. On the source level, the ABA difference between switch and repetition trials in the OCD group is strongest in one cluster of voxels in the the left superior parietal lobule (BA 7), left inferior parietal lobule (BA 40), and left postcentral gyrus (BA 1/2/3) (Fig. 4) and in the HC group in one clusters of voxels in the right superior parietal lobule (BA 7) and right precuneus (BA 7). The significant electrode locations of the cluster-based permutation test are visualized and the time–frequency representation of the power differences between switch and repetition trials can be found in Fig. 3, Fig. 4.

#### 3.2.2 Post-target interval

In the post-target interval (0 ms – 1000 ms) in the cue-based block a cluster-based permutation test revealed a significant negative difference in ABA between the switch and repetition trials (switch < repetition; p = 0.033) in the OCD group, indicating lower ABA in the switch as compared with the repetition condition. On the source level, the ABA difference between switch and repetition trials is strongest in three cluster of voxels: one cluster in the right precuneus (BA 7), one cluster in the left superior parietal lobule (BA 7) and one cluster in the right Precentral (BA 4) and right postcentral gyrus (BA 1/2/3). (Fig. 3). There was no significant effect in the HC group nor in TBA in the cue-based block.

In the memory-based block in the post-target interval (0 ms – 1000 ms) a cluster-based permutation test revealed a significant negative difference between the switch and repetition trials in TBA (switch < repetition) in the OCD group (*p* = 0.02) and HC group (p < 0.005) indicating lower TBA in the switch as compared with the repetition condition. On the source level, the TBA difference between switch and repetition trials is strongest in one cluster in the OCD group: right middle frontal gyrus (BA 46), right inferior frontal gyrus, opercular part (BA 44/45), right inferior frontal gyrus, triangular part (BA 45/46) and two clusters of voxels in the HC group: one cluster is in the left middle temporal gyrus (BA 21) and one cluster in the left inferior parietal lobule (BA 40) and the left supramarginal gyrus (BA 40) (Fig. 4). Additionally, there was a significant negative ABA effect (switch < repetition; p < 0.005) in the HC group in two clusters of voxels: one cluster in the left middle temporal gyrus (BA 21), another in the left middle occipital gyrus (BA 19), and one in the left superior parietal lobule (BA 7) (Fig. 4).

The significant electrode locations of the cluster-based permutation test are visualized and the time–frequency representation of the power differences between switch and repetition trials can be found in Fig. 3, Fig. 4.

To examine the robustness of the anatomical results, we repeated the spatial clustering analysis using different voxel power thresholds. The additional analyses (0.5 % and 2 %) are provided in the Supplementary Material (Table S1 and Table S2). Importantly, the primary anatomical patterns remained consistent across thresholds. As expected, higher thresholds resulted in larger clusters, whereas lower thresholds led to smaller clusters.

## 4 Discussion

The present study examined how adolescents with OCD recruit cognitive control mechanisms during cue-based and memory-based task switching, using behavioral indices and oscillatory (theta- and alpha-band) neural markers. Conceptually, this work contributes to answering the question of why adolescents with OCD often show cognitive rigidity despite equivocal behavioral evidence (Fineberg and Robbins, 2021): namely, whether flexibility deficits emerge specifically when endogenous control, internal monitoring, and working-memory–dependent task-set management are required. Cue-based and memory-based switching provides a meaningful dissociation between externally triggered reactive control and internally generated proactive/monitoring-based control, which is particularly relevant for OCD, where internal guidance and self-initiated adjustments often break down. A second overarching objective was to determine whether theta-band activity (TBA) and alpha-band activity (ABA), which are fundamental to action reconfiguration and gating processes, are differentially modulated in adolescents with OCD across switching contexts. Overall, the results reveal a dissociation between behavioral performance and oscillatory dynamics.

Behaviorally, both groups showed typical switch-cost patterns, and memory-based switching was more demanding than cue-based switching. The OCD group demonstrated lower overall accuracy, aligning with reports of subtle executive inefficiencies in adolescent OCD (Geller et al., 2018), but did not show disproportionate switch costs. The absence of group differences in switch costs suggests that flexible performance per se is not substantially impaired in adolescents with OCD when assessed in a standard behavioral paradigm. While no group-by-condition interactions emerged at the behavioral level, this does not imply equivalent neural mechanisms. Rather, it motivated a neurophysiological approach aimed at characterizing group-specific oscillatory dynamics underlying similar behavioral performance. This aligns with previous studies reporting intact behavioral switching despite neural alterations (Britton et al., 2010, Gu et al., 2007). At the same time, adolescents with OCD showed lower overall accuracy, consistent with broader findings of reduced precision in executive tasks (Geller et al., 2018). The greater difficulty of the memory-based block confirms that endogenous monitoring and working-memory–driven task-set maintenance impose greater cognitive demands. Crucially, Wolff et al.(2017a) reported a memory-specific impairment of cognitive flexibility with no switch costs in the cue-based block. In contrast, in the current study, even the cue-based block exhibited slight switch costs. These differences may be explained by sample size and clinical heterogeneity: despite higher symptom severity in (Wolff et al., 2017a), the larger sample in the present study allowed detection of subtle cue-block effects. Medication had no significant effect, indicating that pharmacological treatment does not account for the observed differences. Notably, the absence of group-by-condition interactions implies that behavioral measures alone underestimate the control disturbances in adolescent OCD, thereby motivating the use of oscillatory measures to uncover subtler neural level changes. Indeed, results of the neural data analyses showed preserved control engagement during externally cued flexibility. While a formal group-by-condition interaction was not performed at the source level, the present findings reveal robust within-group condition effects that differ in their spatial and oscillatory patterns between groups. This pattern indicates that the two groups may use different neural strategies during task switching. Specifically, selective disruptions during internally guided flexibility in the OCD group point toward an underlying architecture of control that is compromised only when endogenous monitoring is required. This is discussed below:

TBA has been associated withtask-set updating, conflict detection, and reactive control recruitment (Cavanagh and Frank, 2014, Cohen, 2014, Cooper et al., 2016, Sauseng et al., 2006). In the cue-based block, both groups demonstrated robust pre-target increases in TBA during switch versus repetition trials, with source-localized effects in middle and superior frontal regions. This indicates that early preparatory gating processes in cue-based task switching remain intact in adolescent OCD. As these findings resonate with the view that external cues can scaffold performance in OCD (Bednarek et al., 2024, Stern et al., 2017), reducing reliance on internal monitoring. Complementary to TBA (Beste et al., 2023), ABA is thought to serve as a mechanism of attentional gating, inhibition of competing representations, and stabilization of task sets (Bonnefond and Jensen, 2012, Foxe and Snyder, 2011, Klimesch, 2012, Konjusha et al., 2023, Yu et al., 2024). Consistent with these functions, pre-target ABA differentiated switch from repetition trials in both groups and in both switching modes. While pre-target Alpha-Theta coordinated activity was shown in HC in both blocks, it was only seen in the OCD group in the cue-based block. The memory-based block revealed a group-related dissociation: only neurotypical adolescents showed switch-related pre-target increases in TBA, whereas adolescents with OCD did not. This dissociation is not interpreted as a group-by-condition interaction in a factorial sense, but as evidence for distinct neural implementations of internally guided flexibility in the two groups. Given that memory-based switching requires participants to internally track rule transitions and generate task-set updates without external signals (Petruo et al., 2021, Wolff et al., 2017b), the absence of TBA modulation in the OCD group may suggest a deficit in endogenous reconfiguration processes. This aligns with conceptual frameworks that posit that TBA is necessary for action reconfiguration (Beste et al., 2023), with findings that OCD involves difficulties in internally maintained representations and self-initiated adjustments (Olley et al., 2007, Snyder et al., 2015), and with empirical evidence of working-memory vulnerabilities in pediatric OCD (Geller et al., 2018). The localized frontal clusters in neurotypical adolescents further support the notion that medial superior frontal regions orchestrate internal control (Li et al., 2013, Niendam et al., 2012, Tang et al., 2016). In contrast, adolescents with OCD fail to recruit this network when external guidance is absent appropriately.

However, post-target dynamics revealed group differences in cue-based switching with the OCD group displaying switch-related ABA reductions in centro–parietal regions, whereas HCs showed no such effect. These reductions may indicate insufficient task-set stabilization after stimulus onset, reflecting abnormalities in how OCD adolescents use previously gated information following stimulus processing. This interpretation is consistent with findings that alpha supports network stabilization and the protection of ongoing task states (Elmers et al., 2024, Klimesch, 2012, Konjusha et al., 2023, Yu et al., 2024).

In the memory-based block, HCs exhibited coordinated TBA and ABA differentiation post-target–reflecting an integrated oscillatory response that supports internally generated updating and gating. In contrast, adolescents with OCD showed only TBA modulation, indicating preserved updating signals but no accompanying ABA modulation, and thus no coordinated TBA-ABA pattern. This suggests a reduced capacity to orchestrate flexible control when task switching relies on internal task-set management. The frontal cluster observed in the OCD group likely reflect compensatory or inefficient gating processes that fail to integrate with TBA-mediated updating.

These findings thus reveal a clear dissociation between externally and internally guided flexibility. In cue-based switching, behavior remained intact and both TBA and ABA modulation were preserved. In contrast, memory-based switching was more demanding for all participants. While adolescents with OCD showed only post-target TBA modulation without accompanying ABA modulation and thus no coordinated TBA-ABA pattern, indicating impaired internally driven updating and a reduced capacity to orchestrate flexible control. These dynamics further underscores that adolescents with OCD rely on altered temporal control mechanisms to achieve comparable behavioral outcomes, particularly under externally guided conditions. This dissociation helps explain why cognitive rigidity in OCD may manifest more strongly in real-world situations that rely on self-initiated, sustained, and internally monitored control rather than on externally structured tasks (Bednarek et al., 2024, Gillan et al., 2016, Lavallé et al., 2020, Lazarov et al., 2010). We note that all participants completed the blocks in the same fixed order, which could introduce practice or order effects. Because no alternative order was tested, we cannot fully rule out such effects. However, the main findings were consistent across participants within this fixed sequence.

In summary, we show that adolescents with OCD can flexibly update behavior when external cues clearly signal task demands, but they struggle when flexible adjustments must be generated internally. In the cue-based block, both behavioral performance and oscillatory markers of control were broadly comparable across groups, indicating that exogenous structure supports the recruitment of reactive control mechanisms. In contrast, the memory-based block revealed group differences at the neural level: Although early preparatory ABA modulation was intact, later-stage processes involved in stabilizing the new task set were disrupted and also lacked the coordinated TBA-ABA pattern seen in HC adolescents. These results suggest that the core difficulty in adolescent OCD lies in initiating and organizing cognitive control when no external signals are available, providing a possible mechanistic explanation for why rigidity becomes especially apparent in everyday situations that rely on internal monitoring and self-guided adjustments. Crucially, these findings highlight that comparable behavioral outcomes can be supported by fundamentally different neural control architectures, underscoring the importance of pattern-based neurophysiological interpretations beyond traditional factorial group comparisons.

## CRediT authorship contribution statement

**Sarah Rempel:** Writing – review & editing, Writing – original draft, Visualization, Validation, Software, Project administration, Methodology, Investigation, Formal analysis, Data curation, Conceptualization. **Adriana Böttcher:** Writing – review & editing, Visualization, Validation, Software, Formal analysis. **Nicole Beyer:** Writing – review & editing, Supervision, Resources, Methodology, Investigation, Conceptualization. **Veit Roessner:** Writing – review & editing, Funding acquisition. **Christian Beste:** Writing – review & editing, Supervision, Software, Resources, Methodology, Funding acquisition, Conceptualization.

## Data Availability

Data will be made available on request.
