# Supplementary material for: Theta–Alpha Dysregulation reveals impaired endogenous cognitive control in adolescent Obsessive–Compulsive disorder
Source: Neuroimage Clin. 2026 Feb 20;49:103975. doi: 10.1016/j.nicl.2026.103975 (PMC12945646; doi:10.1016/j.nicl.2026.103975)
Supplement: Supplementary Data 1 [file mmc1.docx]

**Supplemental Material**

**Theta–Alpha Dysregulation Reveals Impaired Endogenous Cognitive Control in Adolescent Obsessive–Compulsive Disorder**

Sarah Rempel, Adriana Böttcher, Nicole Beyer, Veit Roessner, Christian Beste

*Comorbid diagnoses and medications*

Table S1: Description of comorbid diagnoses and medications. There are 6 individuals with combinations of comorbid diagnoses and 3 individuals with combinations of medications.

| **ICD-10 Code** | **Count** | **Medication** | **Count** |  |
| --- | --- | --- | --- | --- |
| F95.2 | 6 | Fluoxetine | 11 |  |
| F32.1 | 3 | Sertraline | 6 |  |
| F40.1 | 3 | Fluvoxamine | 4 |  |
| F90.0 | 2 | Risperidone | 1 |  |
| F50.1 | 2 | Aripiprazole | 1 |  |
| F40.0 | 2 | Agomelatine | 1 |  |
| F81.2 | 1 | Medikinet | 1 |  |
| F63.3 | 1 | Methylphenidate | 1 |  |
| F43.2 | 1 | Escitalopram | 1 |  |
| F64.9 | 1 | Citalopram | 1 |  |
| F98.88 | 1 | L-Thyroxine | 1 |  |
| F90.1 | 1 |  |  |  |
| F41.8 | 1 |  |  |  |
| F95.1 | 1 |  |  |  |
| F81.0 | 1 |  |  |  |
| F41.0 | 1 |  |  |  |
| F98.8 | 1 |  |  |  |
| F64.0 | 1 |  |  |  |
| F32.2 | 1 |  |  |  |
| F33.1 | 1 |  |  |  |
| F60.31 | 1 |  |  |  |

Medication did not influence switch costs or block-dependent effects in the OCD group, *F*(1,66) = 1.97, *p* = .166, *η²* = .029.

*HCs data sets*

We included in the HCs sample N = 52 unpublished data sets and N = 22 data sets from Wolff et al. (Wolff et al., 2017). The new sample did not differ from the Wolff et al. sample regarding the effect of “Block” (cue vs. memory) or its interaction with condition (repetition vs. switch), all p > .083. To increase statistical power and match the sample size to the OCD group, we therefore used a final HC sample of N = 74.

*Voxel power thresholds*

To examine the robustness of the anatomical results, we repeated the spatial clustering analysis using different voxel power thresholds.

Table S2. Spatial clustering results at voxel power threshold = 0.05%

| **OCD group** | | | |
| --- | --- | --- | --- |
|  | | **CUE-based** | **MEM-based** |
| **Pre-target** | **Theta** | Cluster 1:  030: Frontal_Sup_L | - |
|  | **Alpha** | All clusters < 10 voxels | Cluster 2:  021: Parietal_Sup_L |
| **Post-target** | **Theta** | - | Cluster 1:  025: Frontal_Mid_R  012: Frontal_Inf_Oper_R |
|  | **Alpha** | Cluster 2:  010: Postcentral_R | - |
| **HC group** | | | |
| **Pre-target** | **Theta** | Cluster 1:  013: Frontal_Mid_Orb_L  Cluster 4:  010: Frontal_Sup_R | Cluster 1:  012: Frontal_Sup_Medial_R |
|  | **Alpha** | Cluster 1:  018: Occipital_Mid_L | Cluster 1:  022: Parietal_Sup_R  014: Precuneus_R |
| **Post-target** | **Theta** | - | Cluster 2:  025: Parietal_Inf_L |
|  | **Alpha** | - | Cluster 1:  034: Temporal_Mid_L |

Table S2. Spatial clustering results at voxel power threshold = 2%

| OCD group | | | |
| --- | --- | --- | --- |
|  | | **CUE-based** | **MEM-based** |
| Pre-target | **Theta** | Cluster 3:  042: Frontal_Sup_L  033: Frontal_Sup_Medial_L  030: Frontal_Mid_L  027: Frontal_Sup_R  015: Frontal_Sup_Medial_R | - |
|  | **Alpha** | Cluster 2:  034: Precuneus_L  023: Occipital_Mid_L  018: Angular_L  015: Parietal_Sup_L  014: Occipital_Sup_L  011: Parietal_Inf_L | Cluster 3:  071: Parietal_Sup_L  031: Parietal_Inf_L  029: Postcentral_L  017: Occipital_Mid_L  017: Occipital_Sup_L |
| Post-target | **Theta** | - | Cluster 3:  081: Frontal_Mid_R  031: Frontal_Inf_Oper_R  024: Frontal_Inf_Tri_R  017: Precentral_R  Cluster 4:  012: Frontal_Mid_L |
|  | **Alpha** | Cluster 1:  032: Precuneus_R  030: Precentral_R  024: Postcentral_R  Cluster 2:  018: Parietal_Sup_L  016: Postcentral_L | - |
| HC group | | | |
| Pre-target | **Theta** | Cluster 1:  027: Frontal_Inf_Tri_L  026: Frontal_Mid_Orb_L  012: Frontal_Inf_Orb_L  Cluster 2:  056: Frontal_Sup_R  022: Frontal_Sup_Medial_R  020: Frontal_Mid_R | Cluster 1:  041: Frontal_Sup_Medial_R  027: Frontal_Sup_R |
|  | **Alpha** | Cluster 1:  073: Occipital_Mid_L | Cluster 1:  065: Precuneus_R  063: Parietal_Sup_R  021: Precuneus_L |
| Post-target | **Theta** | - | Cluster 1:  045: Temporal_Mid_L  034: Parietal_Inf_L  028: SupraMarginal_L  027: Temporal_Sup_L  Cluster 3:  010: Frontal_Sup_R |
|  | **Alpha** | - | Cluster 1:  095: Temporal_Mid_L  020: Occipital_Mid_L  011: Temporal_Inf_L  010: Occipital_Inf_L  Cluster 2:  031: Parietal_Sup_L |
